# Supplementary material for: The Deubiquitinating Enzyme AMSH1 Contributes to Plant Immunity Through Regulating the Stability of BDA1
Source: Plants (Basel). 2025 Feb 1;14(3):429. doi: 10.3390/plants14030429 (PMC11819993; doi:10.3390/plants14030429)
Supplement: Supplementary file 1 [file plants-14-00429-s001.zip › plants-3405173-supplementary.pdf]

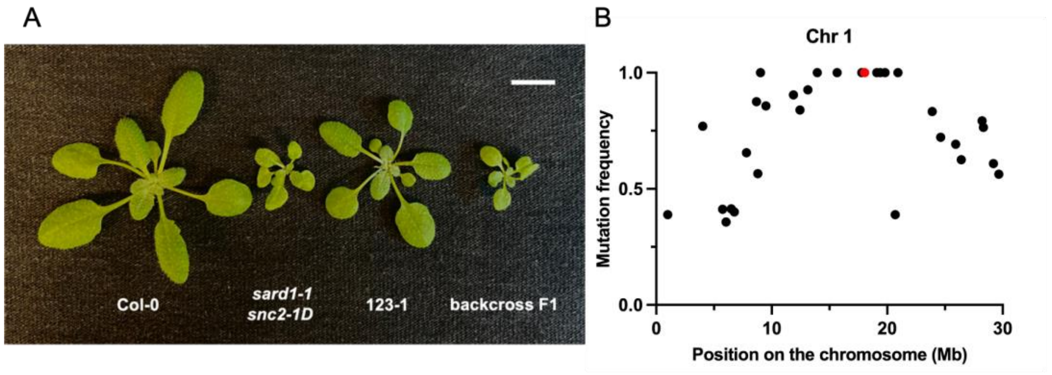

Figure S1. Mapping-by-sequencing of 123-1.

(A) Morphology of 24-day-old soil-grown plants of the indicated genotypes under long-day condition. Scale bar is 1 cm.

(B) Linkage region for the 123-1 mutation in the middle of chromosome 1. The mutation in *AMSH1* is highlighted using a red data point.

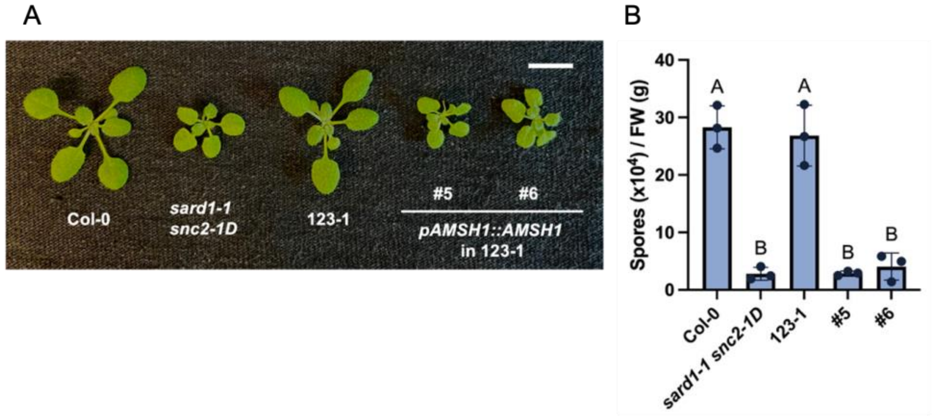

Figure S2. *pAMSH1::AMSH1* complements the phenotypes of the suppressor 123-1.

(A) Morphology of 21-day-old soil-grown plants of the indicated genotypes under long-day condition. #5 and #6 are two independent complementation lines of *AMSH1* in the 123-1 background. Scale bar is 1 cm.

(B) Growth of *Hpa* Noco2 conidiospores on the indicated genotypes. Error bars represent standard deviations. Letters indicate statistical differences ($P < 0.05$, one-way ANOVA; $n = 3$).

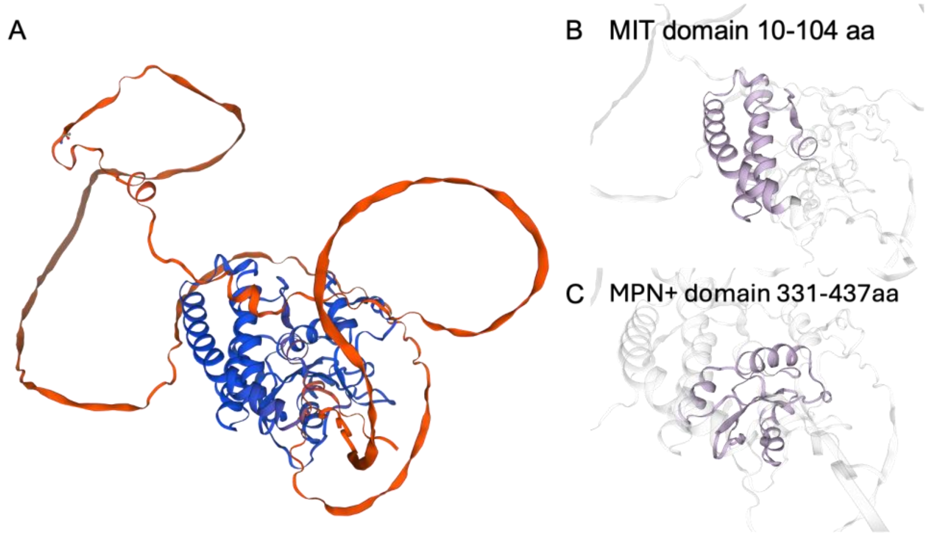

Figure S3. Predicted structure of AMSH1 protein.

(A) Structure of full length AMSH1 protein predicted by AlphaFold. Functional MIT domain and MPN+ domain are labeled with blue color, middle region is labeled with red color. (B) Predicted 3D structure of AMSH1 N terminal MIT domain in purple color. (C) Predicted 3D structure of AMSH1 C terminal MPN+ domain in purple color.

Supplementary Table S1. Primers used in this study.

Primer name	Primer sequence (5′ to 3′)
AMSH1-T-DNA-F	TATGGTTTATCCAACCATGGG
AMSH1-T-DNA-R	TAGTTTCACTTTCAAACGCGC
AMSH1-DT1-F	ATATATGGTCTCGATTGCTTTCCAAGCATTCTATCTGTTTTAGAGCTAGAAATAGC
AMSH1-DT2-R	ATTATTGGTCTCGAAACGTACTTCAAATATCTCTTCCAATCTCTTAGTCGACTCTAC
AMSH1-dele-F	TCAAGCTCATCCAGCAAATG
AMSH1-dele-R	GTGAACTGTGCGCATGAAAC
AMSH1-homo-F	CAACCTTCACCACCTCCAGT
AMSH1-pro-EcoRI-F	GACTGAATTCCTGACAACAGTTACAGGCATCC
AMSH1-StuI-R	GAGAAGGCCTTCTGAGATCAATGACATCAA
Nb-AMSH1-F1	ATTTGGAGAGAACAGAATTCGAGCTCTCCTAAAGTTGAAAATTTG
Nb-AMSH1-R1	TTATTTCCTTACCAATTGGGGTACCCTTGGGACTTGAGACACAAA
Nb-AMSH1-F2	GTTCGAAATCGATAAGCTTGGATCCCTTGGGACTTGAGACACAAA
Nb-AMSH1-R2	TAAGCTTGCATGCCTGCAGTCTAGATCCTAAAGTTGAAAATTTGT
